# Global Forestry Areas, Deforestation and Mental Health: A Worldwide Ecological Study

**DOI:** 10.1192/bjo.2025.10203

**Published:** 2025-06-20

**Authors:** Alice Bolton

**Affiliations:** Barts and The London School of Medicine and Dentistry, London, United Kingdom

## Abstract

**Aims:** Forests are important for planetary and human health, but deforestation is increasing. Poor mental health is increasingly affecting the world’s population. This study aims to investigate the association between forestry area, deforestation and mental health, at country level, worldwide.

**Methods:** Forestry area in each country was sampled in 2006 and 2016; the country prevalence of mental health disorders or substance abuse was sampled in 2006 and 2016; and the relative disability-adjusted life years (DALYs) in 2010 and 2016. Crude and multivariate linear regression analyses were run, adjusting for peace index, wealth and inequalities, and urbanisation at country level. A sensitivity analysis including sanitation and food security was run. Interaction with country gross domestic product per capita was assessed.

**Results:** Based on data for 230 countries, country forestry area is negatively associated with the prevalence of mental health disorders in 2016 (β −0.02 (195% C.I. −0.04/−0.01). This association was maintained in sensitivity analyses, and found mainly in lower- and upper-middle income countries. Change in forestry area is not associated with mental health prevalence nor estimated DALYs due to mental health.

**Conclusion:** This is the first study showing that forestry area at country level is associated with a lower prevalence of mental health disorders. If these results are replicated at individual level, this would suggest that public health implications should play a strong role in weighting ecological decisions, such as optimising forestry area coverage.

